# Identification and Quantitation of Asparagine and Citrulline Using High-Performance Liquid Chromatography (HPLC)

**Published:** 2007-03-28

**Authors:** Cheng Bai, Charles C. Reilly, Bruce W. Wood

**Affiliations:** United States Department of Agriculture, Agricultural Research Service, South Atlantic Area, Southeast Fruit and Tree Nut Research Laboratory, 21 Dunbar Road, Byron, Georgia 31008.

**Keywords:** HPLC identification, pecan, ureides, asparagine, citrulline

## Abstract

High-performance liquid chromatography (HPLC) analysis was used for identification of two problematic ureides, asparagine and citrulline. We report here a technique that takes advantage of the predictable delay in retention time of the co-asparagine/citrulline peak to enable both qualitative and quantitative analysis of asparagine and citrulline using the Platinum EPS reverse-phase C18 column (Alltech Associates). Asparagine alone is eluted earlier than citrulline alone, but when both of them are present in biological samples they may co-elute. HPLC retention times for asparagine and citrulline were influenced by other ureides in the mixture. We found that at various asparagines and citrulline ratios [= 3:1, 1:1, and 1:3; corresponding to 75:25, 50:50, and 25:75 (μMol ml^−1^/μMol ml^−1^)], the resulting peak exhibited different retention times. Adjustment of ureide ratios as internal standards enables peak identification and quantification. Both chemicals were quantified in xylem sap samples of pecan [*Carya illinoinensis* (Wangenh.) K. Koch] trees. Analysis revealed that tree nickel nutrition status affects relative concentrations of Urea Cycle intermediates, asparagine and citrulline, present in sap. Consequently, we concluded that the HPLC methods are presented to enable qualitative and quantitative analysis of these metabolically important ureides.

High-performance liquid chromatography (HPLC) is a potentially powerful tool for quantitative and qualitative analysis of substances possessing similar chemical characteristics. While varying column types, column conditions, and eluate characteristics generally enable peak resolution sufficient for fractionation and analysis of similar chemical constituents in samples, such manipulations are often unsuccessful for ureide mixtures containing both asparagine and citrulline. Ureides are cyclic or acyclic acyl derivatives of urea and play key role in nitrogen metabolism and cycling in certain species of higher plants. Qualitative and quantitative analysis of these two ureides is critical for studies assessing Urea Cycle activity, a metabolic cycle involving nitrogen cycling in organisms, and factors affecting cycle functionality.

Most reversed-phase silica-based media are covalently bonded. The Platinum EPS phase is unique for HPLC analysis based on the utilization of the interactions of the underlying base silica to enhance the selectivity of polar organic analysis (Alltech Associate, Inc. 2002). The Platinum EPS reversed-phase C18 column (Alltech Associates) has been used for ureide analysis (Bai et al. 2006), although its utility is limited in that analysis of asparagine and citrulline, both key Urea Cycle ureides, is problematic. Ureides can also be analyzed via the ZORBAX Rx-SIL (Agilent Technologies) column (made from porous silica microspheres). It is used for normal-phase chromatography and is designed for high stability at low pH (Agilent Technologies, 2006), but was found by the authors to be inferior to the Platinum EPS column for separation of asparagine and citrulline.

Both asparagine and citrulline are considered as nonessential amino acids. Asparagine is one of the 20 most common natural amino acids in biology. It has carboxamide as the side chain’s functional group, thus allowing for efficient hydrogen bond interactions with peptide backbones. The precursor to asparagine is oxaloacetate, which is converted to aspartate by a transaminase (Nelson and Cox, 2000). Citrulline is an amino acid critical to the detoxification and elimination of unwanted ammonia within cells. During metabolism, citrulline is a precursor of arginine (Nelson and Cox, 2000). Chemical structures for asparagine and citrulline are as follows:

**Figure:**
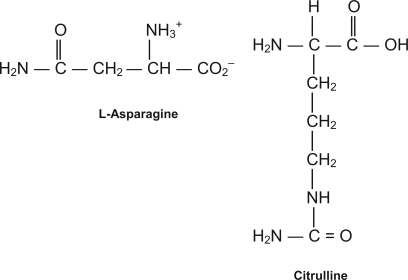

We present here an important yet simple technique that overcomes co-elution problems often encountered with HPLC elution of asparagine and citrulline, thus enabling quantitative and qualitative analysis of L-asparagine and citrulline by HPLC using a Platinum EPS C18 column.

## Experimental Section

### Biological materials and standards

Xylem sap of pecan [*Carya illinoinensis* (Wangenh.) K. Koch] trees were used for ureide analysis. Two classes of trees were assessed, those that were nickel sufficient (Ni-S) and those nickel deficient (Ni-D). Samples were from multiple 3-year-old greenhouse-grown seedling trees exhibiting either of these two Ni nutritional classes. Classification of Ni deficiency was based on presence/absence of morphological symptoms of Ni deficiency and by tissue Ni concentration (Bai et al. 2006). The ureide standards were acquired from commercial sources. L-asparagine anhydrous (99.5%) was purchased from Fluka Biochemika (St. Louis, Mo), whereas citrulline (98%) and other ureides (e.g. allantoic acid and allantoin) were from Sigma (St. Louis, Mo).

### HPLC analysis of ureides

The methods for ureide analysis were similar to those described previously (Bai et al. 2006). Analysis was performed on a Spectra System SCM 1000 HPLC linked with a Spectra System UV 1000 detector (Thermo Electron Corporation, San Jose, CA) using a Platinum EPS C18 column, 5 μm, 250 mm × 4.6 mm (Alltech Associates, Deerfield, IL). Mobile phase was acetonitrile: 0.03 M potassium phosphate, pH 3.2 (20:80). Flow rate was 0.5 ml/min at a column temperature of 30°C. Sample injection volume was 20 μl. Ureides were detected at 190 nm. Tentative ureide identification was based on identical retention times (RT) compared to ureide standards. The standard ureides (asparagine, citrulline, allantoic acid and allantoin) and internal standard ureides (the same as mentioned as above) were used at a range of 10–100 μMoles ml^−1^ [in acetonitrile: 0.03 M potassium phosphate monobasic pH 3.2, (20:80)]. Plant samples were analyzed for five replicates of each Ni treatment class. A ZORBAX Rx-SIL (Agilent Technologies) column (5 μm, 4.6 mm × 150 mm) with a mobile phase consisting of acetonitrile, 20 mM potassium phosphate, pH 7.2 (90:10) was used to verify the ureide analysis using the Platinum EPC column.

### Influence of different ratios of asparagine and citrulline

The influence of asparagine and citrulline ratio on retention time was evaluated using either 100 μMol ml^−1^ total concentration. The concentrations of asparagine and citrulline were 75:25, 50:50, and 25:75 (μMol ml^−1^:μMol ml^−1^), corresponding to the ratios of 3:1, 1:1, and 1:3. Analysis was performed using the Platinum EPS C18 column. The protocol typically resulted in very small differences in retention times for asparagine and citrulline (5.84 min and 5.87 min, respectively) when analyzed as a mixture at equivalent concentrations of allantoic acid, asparagine, citrulline and allantoin. A mixture of these four ureides was then analyzed as above but at different ureide ratios of 1:7:1:1 and 1:1:7:1 of allantoic acid: asparagine: citrulline: allantoin solutions (total concentrations: 100 μMol ml^−1^). In the present study, we added a relatively high concentration of either asparagine or citrulline as a carrier to the plant sample; thus, the retention time for either citrulline or asparagine in the mixed sample is essentially the same as that for the standard alone. It is noteworthy that the influence on the retention times for asparagine and citrulline diminishes, and resulting peaks are resolved if several ureides are mixed for HPLC analysis. The amount of either asparagine or citrulline in the biological sample is then determined by subtracting the peak area of “a single standard of another run” from the relatively large peak area obtained from a run containing both a “biological sample plus a single standard”. The calculation is as follows:

(1) $$\begin{array}{c} \text{S}\;=\;\text{the}\;\text{peak}\;\text{area}\;\text{of}\;\text{ureide}\\ (\text{i}.\text{e}.\;\text{asparagine}\;\text{or}\;\text{citrulline})\;\text{standard} \end{array}$$

Note that the value of S is determined after a standard is run alone.

(2) $$\text{A}\;=\;\text{M}\;-\;{\text{S}}_{2}$$

Where A = the peak area of the ureide in the biological sample;

M: = the peak area of combined mixture of sample and ureide standard (S_1_); and
S_2_: = the peak area of the ureide standard alone, determined in a separate HPLC run.

Note that the combined mixture of the respective ureide standard at a relatively high concentration (S_1_) and the respective ureide from biological sample results in a relatively large peak area (M) as compared with other peak area, which are relatively small. S_1_ and S_2_ are analyzed at equal concentrations of the standard ureide in both runs.

### Preparation of xylem sap fractions

Xylem sap samples were collected from several Ni-D and Ni-S trees at bud break in late March. Sap was collected via vacuum extracted from stems severed about 2 cm above the root collar and again just below the apical tip. Phloem and bark were removed for the distance of 1 cm from the base, and the base of severed stems placed in a vacuum chamber, the exuding xylem sap collected into 2 ml vials, and the sap samples immediately frozen.

Xylem sap samples from twelve representative trees of each of the two Ni treatments were randomly selected and then processed in triplicate for reduced-N and urease analysis. Equivalent volumes of xylem sap for each sample were thawed and twice centrifuged (20,000*_g_*) for 30 min. The supernatant was further purified by removing molecules ≥10-kD by twice filtering through a Centricon-10 filter (Millipore filter units, Millipore, Bedford, MA) after centrifugation (5,000*_g_*) for 75 min. The partially purified samples were then analyzed for ureides using UV spectroscopy and HPLC. For identification purposes, samples were processed on the two above described HPLC columns with different eluants.

### Statistical analyses

Concentrations of organic molecules in Ni-D and Ni-S samples were square root transformed, prior to analysis. Differences in concentrations in two classes were compared by t-test (*P* ≤ 0.05; SAS 2001). Mean values were analyzed with four tissue replicates.

## Results and Discussion

### Initial analysis of asparagine and citrulline mixture

**Figure 1.**
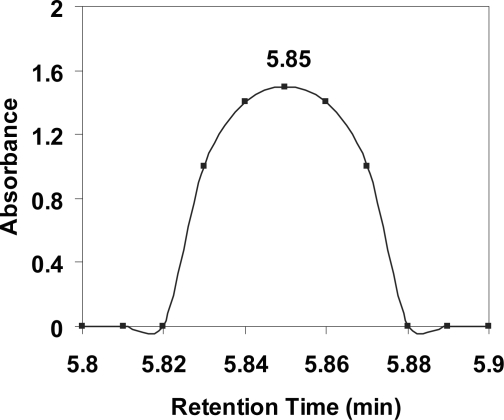
High-performance liquid chromatography (HPLC) separation of a high concentration of 1:1 mixture (100 μMol ml^−1^:100 μMol ml^−1^) of asparagine and citrulline using a Platinum EPS C18 column (5 μm, 250 mm × 4.6 mm) at attenuation 16 and detected at 190 nm. Mobile phase was acetonitrile: 0.03 M potassium phosphate, pH 3.2 (20:80); flow rate was 0.5 ml/min; column temperature was 30°C; and sample injection volume was 20 μl.

HPLC analysis, using the above described Platinum EPS C18 column and elute conditions, of a mixture of asparagine and citrulline yields a single peak reflecting co-elution of asparagine and citrulline (5.85 min) (Figure 1). The ZORBAX Rx-SIL column enabled slight separation of these two ureides, giving only slight differences in retention times (5.41 min for asparagine and 5.44 min for citrulline). Also, one other ureide, allantoic acid, elutes at essentially the same time (5.40 min) as asparagine and citrulline. Thus, asparagine and citrulline analysis using the ZORBAX Rx-SIL column is problematic, especially in the presence of allantoic acid. Thus, the ZORBAX column is unsuitable for quantitative analysis using the above described conditions.

### Analysis of asparagine and citrulline mixture

**Figure 2:**
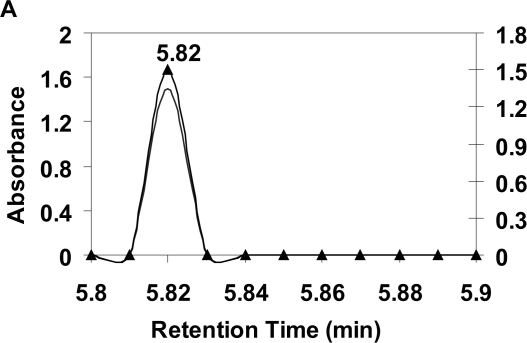

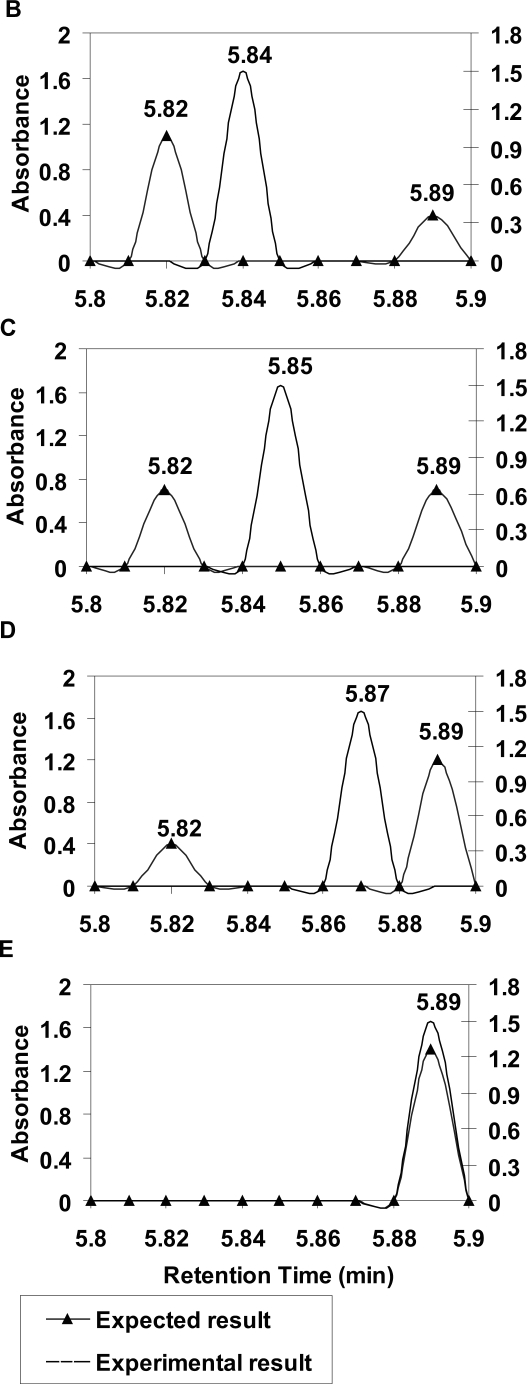
HPLC separation of mixed asparagine and citrulline at various concentration ratios using a Platinum EPS C18 column. Ratios are (**A**) 4:0 (100 μMol ml^−1^:0; asparagine alone); (**B**) 3:1 (75 μMol ml^−1^:25 μMol ml^−1^); (**C**) 1:1 (50 μMol ml^−1^:50 μMol ml^−1^); (**D**) 1:3 (25 μMol ml^−1^:75 μMol ml^−1^); and (**E**) 0:4 (0:100 μMol ml^−1^; citrulline alone) at attenuation 32 and detected at 190 nm. The mobile phase and conditions are as described in Figure 1.

Retention times of eluting ureides generally change with concentration of the target molecule, eluting pH, temperature, and mobile phase composition. While the most common ureides likely to be found in biological samples were marginally resolvable using the two HPLC protocols described above, asparagine and citrulline analysis remained problematic, as shown in the chromatograms (Figure 2). Ureide separation using the Platinum EPS C18 column was such that the retention time for asparagine alone is earlier (Figure 2A) than the mixtures of asparagine and citrulline (Figure 2B, 2C and 2D) and citrulline alone (Figure 2E). Asparagine alone is eluted at 5.82 min (Figure 2A), whereas citrulline alone eluted at 5.89 min (Figure 2E). Different retention times indicate that they satisfactorily separate, thus giving the initial appearance of being an efficacious means of analyzing the two problematic ureides while enabling resolution of other ureides, such as allantoic acid (5.44 min), allantoin (5.93 min), and uric acid (6.06 min) (Bai et al. 2006). However, when asparagine and citrulline are mixed at different ratios the peaks fail to resolve and retention time of the single co-eluting peak slows as citrulline concentration increases (Figure 2). For example: retention times progressively increase to 5.84, 5.85 and 5.87 min, respectively, for a 3:1, 1:1 and 1:3 asparagine/citrulline mixture (Figure 2B, 2C, and 2D). The co-elution is obviously due to an interaction between asparagine, citrulline, and the column, making it difficult to assess results.

**Figure 3:**
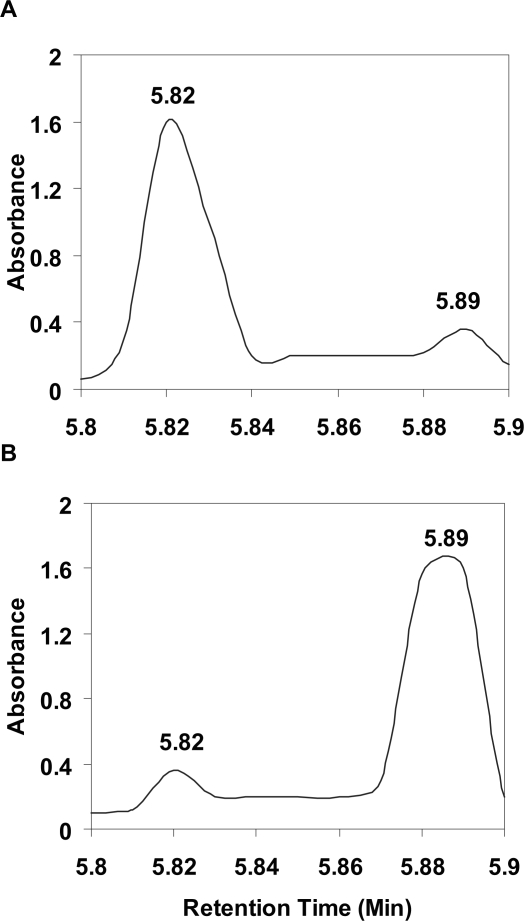
HPLC separation of asparagine and citrulline from a mixture of four ureides at two concentration ratios using a Platinum EPS C18 column. Asparagine was separated from citrulline at ratios of (**A**) 1:7:1:1 and (**B**) 1:1:7:1 (allantoic acid: asparagine: citrulline: allantoin; total concentrations were 100 μMol ml^−1^) at attenuation 16 and detected at 190 nm. The mobile phase and conditions are as described in Figure 1.

Peak symmetry was maintained for all ureide ratio mixtures. It is noteworthy that retention times on the Platinum EPS C18 column increase as the asparagine and citrulline ratios decrease; thus reflecting a complex interaction between these two ureides, the column mediums, and eluting conditions. One possible explanation is that the ureide aggregates arising from the various ratio mixes (e.g. 3:1, 1:1 and 1:3 ratios) differ in the nature of weak chemical interactions associated with hydrogen bond interaction (Nelson and Cox, 2000). Ureide separation using the Platinum EPS C18 column was improved with ureide ratios of 1:1:7:1 and 1:7:1:1 (allantoic acid: asparagine: citrulline: allantoin), with asparagine clearly separating from citrulline (Figure 3). This indicates a reduction in the physiochemical interaction between asparagine and citrulline that otherwise caused co-elution problems for asparagine and citrulline.

The lengthening of asparagine retention time and shortening of citrulline retention time, respectably, within mixtures of ureides is proportional to the relative concentration of these two ureides. However, the influence of citrulline in a sample on asparagine retention time is minor if an additional amount of asparagine is added to the test sample at a relatively high concentration. Similarly, the influence of asparagine on citrulline retention time is minor if an additional amount of citrulline is added to the test sample at a relatively high concentration. When a plant sample is analyzed, analysis is performed using either individual asparagine or citrulline as the major reference for identification with both the Platinum EPS C18 column and the ZORBAX Rx-SIL column. This influence on retention time diminishes if the sample contains several ureides (allantoic acid, allantoin, asparagine, citrulline and uric acid).

### Analysis of asparagine and citrulline in xylem sap of trees with different Ni nutritional status

Pecan trees experience a nickel deficiency in certain growth environments (Wood et al. 2004). Comparative study of Urea Cycle intermediates was made of xylem sap extracts from Ni-S vs. Ni-D trees using the two HPLC columns and methods described above. The identification of these two ureides has been carried out with the Platinum EPS C18 column and the ZORBAX Rx-SIL column. But the concentration of these two ureides listed in Table 1 is derived from the Platinum EPS C18 column. Analysis of the partially purified ≤10 kDa fractions found that asparagine concentration was the highest in spring xylem sap of Ni-S trees, a 78-fold difference in Ni-D trees and that citrulline level was the highest in Ni-D trees, a 57-fold difference in concentration (Table 1). These results identified substantial differences in asparagine and citrulline concentrations in pecan xylem sap. Similar differences were previously noted in pecan foliage (Bai et al. 2006), where asparagine is at a relatively low concentration and citrulline is at relatively high concentration for both Ni-D and Ni-S classes. A substantially greater level (about 1.4-fold) of citrulline was found in foliage of Ni-D trees than that of Ni-S trees (Bai et al. 2006).

In conclusion, we provide a useful means of analyzing asparagine and citrulline in biological samples using HPLC analysis, and present an example of two disrupting ureides interacting with HPLC columns to potentially cause problems with analysis of asparagine or citrulline. We also show that nickel nutritional status can quantitatively affect Urea Cycle intermediates in pecan trees.

## Figures and Tables

**Table 1:** High-performance liquid chromatography (HPLC) separation of asparagine and citrulline from xylem sap of young pecan trees differing in nickel nutritional status.^a^

**Sample type/Ureide**	**(Min)**	**X ± Std^b^ (μMol ml^−1^)**	
		**Ni-deficient**	**Ni-sufficient**
Asparagine	5.82	≤0.5b	39.2 ± 4.7a
Citrulline	5.89	28.7 ± 0.3a	≤0.5b

a Separation was obtained using an Alltech Associates Platinum EPS C18 column. Ni-S = nickel sufficient. Ni-D = nickel deficient.

b Means (± Std) followed by different letters are significantly different (t-test, *P* ≤ 0.05).
